# Ocean plastic crisis—Mental models of plastic pollution from remote Indonesian coastal communities

**DOI:** 10.1371/journal.pone.0236149

**Published:** 2020-07-28

**Authors:** Anna (Anya) Phelan, Helen Ross, Novie Andri Setianto, Kelly Fielding, Lengga Pradipta

**Affiliations:** 1 Business School, Faculty of Business Economics and Law, The University of Queensland, Brisbane, Queensland, Australia; 2 School of Agriculture and Food Sciences, Faculty of Science, The University of Queensland, Brisbane, Queensland, Australia; 3 Faculty of Animal Science, Jenderal Soedirman University, Purwokerto, Central Java, Indonesia; 4 School of Communications and Arts, Faculty of Humanities and Social Sciences, The University of Queensland, Brisbane, Queensland, Australia; 5 Division of Human Ecology, Research Center for Population, Indonesian Institute of Sciences (LIPI), Jakarta, West Java, Indonesia; University of Toronto, CANADA

## Abstract

The crisis facing the world’s oceans from plastics is well documented, yet there is little knowledge of the perspectives, experiences and options of the coastal communities facing overwhelming quantities of plastics on their beaches and in their fishing waters. In emerging economies such as those in the Coral Triangle, the communities affected are among the poorest of their countries. To understand the consequences of ocean plastic pollution in coastal regions, through the eyes of local people, this study examines the knowledge, use, disposal and local consequences of single use plastics in remote island communities in two archipelagos of southern Sulawesi, Indonesia. Using mixed methods—a survey of plastic literacy and behaviour, household interviews about purchasing and disposal, and focus group discussions to generate shared mental models—we identify a complex set of factors contributing to extensive plastic leakage into the marine environment. The rising standard of living has allowed people in low resource, remote communities to buy more single-use plastic items than they could before. Meanwhile complex geography and minimal collection services make waste management a difficult issue, and leave the communities themselves to shoulder the impacts of the ocean plastic crisis. Although plastic literacy is low, there is little the coastal communities can do unless presented with better choice architecture both on the supply side and in disposal options. Our results suggest that for such coastal communities improved waste disposal is urgent. Responsible supply chains and non-plastic alternatives are needed. Producers and manufacturers can no longer focus only on low-cost packaged products, without taking responsibility for the outcomes. Without access to biodegradable, environmentally friendly products, and a circular plastic system, coastal communities and surrounding marine ecosystems will continue to be inundated in plastic waste.

## Introduction

Plastic waste continues to inundate the world’s oceans leading to environmental, economic and social impacts [1, 2]. Millions of tonnes of the plastic waste leaks out of the global consumer market and into the ocean each year [3, 4]. Due to its persistence, durability, and volume, marine plastic debris is harmful not only to marine ecosystems and wildlife, but also to humans [1, 3, 5]. The majority of marine plastic results from land-based sources [3]. Plastic waste generated in coastal regions, that is ineffectively managed, is most at risk of entering the marine environment [6]. While the majority of global plastics waste is generated in the Global North, a large portion of manufacturing of single-use plastic packaging has shifted to Asia [7]. Meanwhile the rising standard of living in the fast growing economies of Southeast Asia, including Indonesia [8], has allowed people in low resource remote communities to buy more single-use plastic items than they could before [9]. Infrastructure for waste management and disposal, however, has not kept up [10, 11].

The proliferation of marine plastic has been found to have a significant negative impact on the function of all marine ecosystem services [1, 2, 5, 12], with consequences for people’s livelihoods in the countries most affected [10, 13]. Well known impacts include ingestion [14–17], entanglement [12, 18, 19], chemical contamination [20], dispersal of invasive species [21], and release of persistent, bio-accumulating and toxic substances (PBTs) [1, 7]. Estimates from one study show that marine plastic pollution reduces the provision of ecosystem services linked to fisheries, aquaculture, recreation and heritage values by 1–5%, resulting in significant economic consequences (with losses roughly estimated between $3,300 and $33,000 per tonne of marine plastic per year) [1]. Another study [22] shows the economic impact of marine debris deterring visits to beaches. Coastal and island communities are particularly vulnerable since they depend directly on healthy marine ecosystems for food, livelihoods, income, cultural, recreational, and spiritual needs [23–25].

The scientific community is only beginning to understand the sources, distribution, and impacts of plastics in the marine environment [6, 26, 27]. Additional information is needed at local and regional scales to understand intricate causation and develop appropriate management capability and mitigation strategies [28]. This requires, better understanding of factors contributing to marine litter and unmanaged plastic waste in under-researched regions outside urban population centres, such as remote and coastal communities. The dynamic nature of the problem, including understanding the decisions made by value chain actors including the producers, users and disposers of the products packaged in plastics that lead to ocean plastic pollution, requires system thinking [29, 30].

In low-income developing countries the rate of marketing and distribution of Fast Moving Consumer Goods (FMCG) continues to grow [31]. Throughout Asia, the resultant plastic waste is exacerbated by single-use ‘sachet’ size product distribution directed towards low socio-economic communities and low-income families who buy most of their food in small daily portions [32–35]. Sachets are single-use packets made of plastic and aluminium that form a large portion of the FMCGs market [36, 37]. Sachet packaging is notoriously difficult to recycle [38] and is particularly prevalent in remote and rural communities which have less sophisticated waste management infrastructure [37].

Inadequate waste management, combined with population growth and economic factors, is understood to affect plastic accumulation trends [3]. Littered and inadequately disposed waste, including open dumps and uncontrolled landfills, contribute to plastic waste leakage into the marine environment via waterways, winds and tides [39]. The adequacy of waste collection and recycling infrastructure varies greatly between regions and countries. While high-income countries generate more plastic waste per capita, most have effective waste collection systems [3]. Many middle and low income countries lack both effective waste collection and waste management systems, resulting in these countries being the main sources of global plastic pollution [39]. Globally, recycling rates for plastic are low, with only an estimated 14% of plastic packaging collected for recycling [40]. Furthermore, exports of recyclable materials from developed to developing countries has resulted in significant transfer of waste pollution [41, 42].

The social and economic costs of plastic wastes are often borne by those affected rather than those responsible [43, 44]. Strategies to reduce plastic waste pollution are typically targeted at consumers [45], with a focus on behaviour change [46, 47] to the exclusion of other potential intervention points in this complex system. There is some attention to improving waste management in densely populated urban centres in Asia and Indonesia [48–51], but much less is known about the factors contributing to ocean plastic pollution in remote coastal communities, how the local communities are perceiving the plastic problem, and what this could mean for potential solutions.

The purpose of this study is to understand factors contributing to the use and disposal of single use plastics in coastal and island communities in Eastern Indonesia and to offer new insights into unmanaged plastic waste and the support that is required to solve it. We explored community members’ ‘plastic literacy’ to examine the knowledge and understanding of community members about the issues of plastic waste and marine plastic. In addition we sought information on households’ waste generation and disposal practices in more detail, to learn more about behaviour patterns. We elicited community members’ mental models about the causes and effects of plastics in their waters and their lives. Using a systems thinking approach, we explored how these community mental models link factors contributing to ocean plastic pollution with their key livelihoods, such as fisheries, aquaculture, and tourism. In combination, the information suggests a variety of potential intervention points in the system of plastic waste pollution in Indonesia’s more remote waters.

### Locations and background information

Some estimates suggest that the island nation of Indonesia is the second-largest contributor to marine plastic pollution after China [3, 52]. This is important because Indonesia has some of the most biodiverse and important coral reef systems in the world, with the highest diversity of reef fish and coral species [53]. Reef ecosystems, alongside seagrass and mangroves, support livelihoods and food security for millions of people, and provide essential functions that ensure water quality, carbon sequestration and storm surge protection, to name a few [54].

The Indonesian archipelago comprises 17,508 islands, among which 6,000 are inhabited, with 81,000 km of coastline [55]. The country is home to 271.8 million people [56] with 65–70% of the population (approximately 190 million) living near the coast [55, 57]. Previous studies show that uncollected and unmanaged plastic waste, generated within 50 km of the coast, eventually makes its way into the ocean via multiple outlets including rivers [3, 6].

We sought locations that represent typical remote coastal communities in Indonesia. The majority of coastal communities in Indonesia are impacted by both ocean plastic debris transported from other locations, and locally-sourced waste from land-based leakage and disposal in the sea. The criteria for choosing the study sites included: communities that were remote from commercial centres or urban population; close to sensitive marine areas, (e.g. areas rich in coral reefs and biodiversity); subject to seasonal storms and thus plastic marine debris; and communities with minimal tourism. Note that the majority of the remote, coastal communities in Indonesia do not have extensive tourism. Areas with tourism tend to have more organised beach clean-ups, as proprietors initiate waste mitigation efforts with or without involving the local communities.

**Fig 1:**
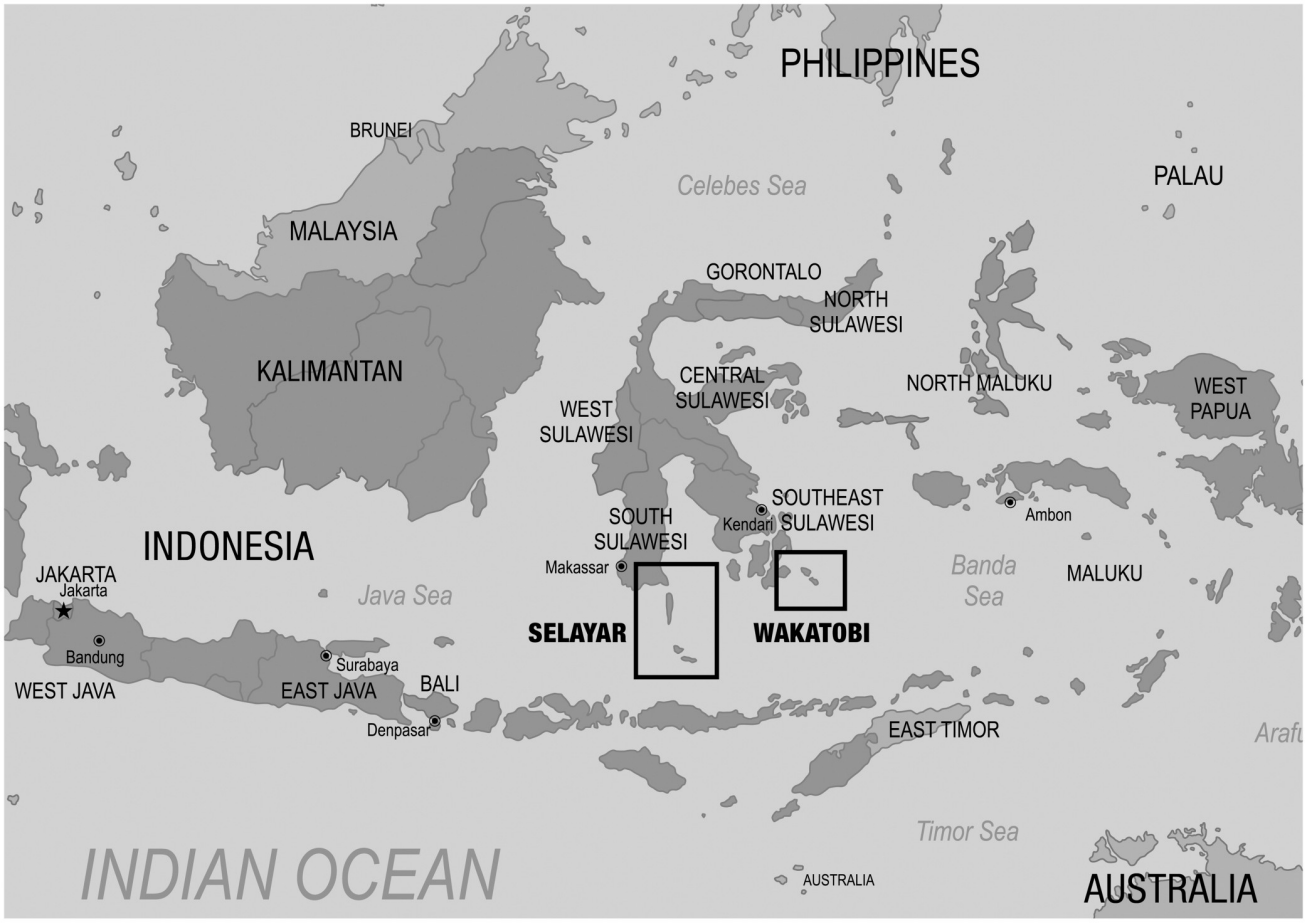


**Fig 2:**
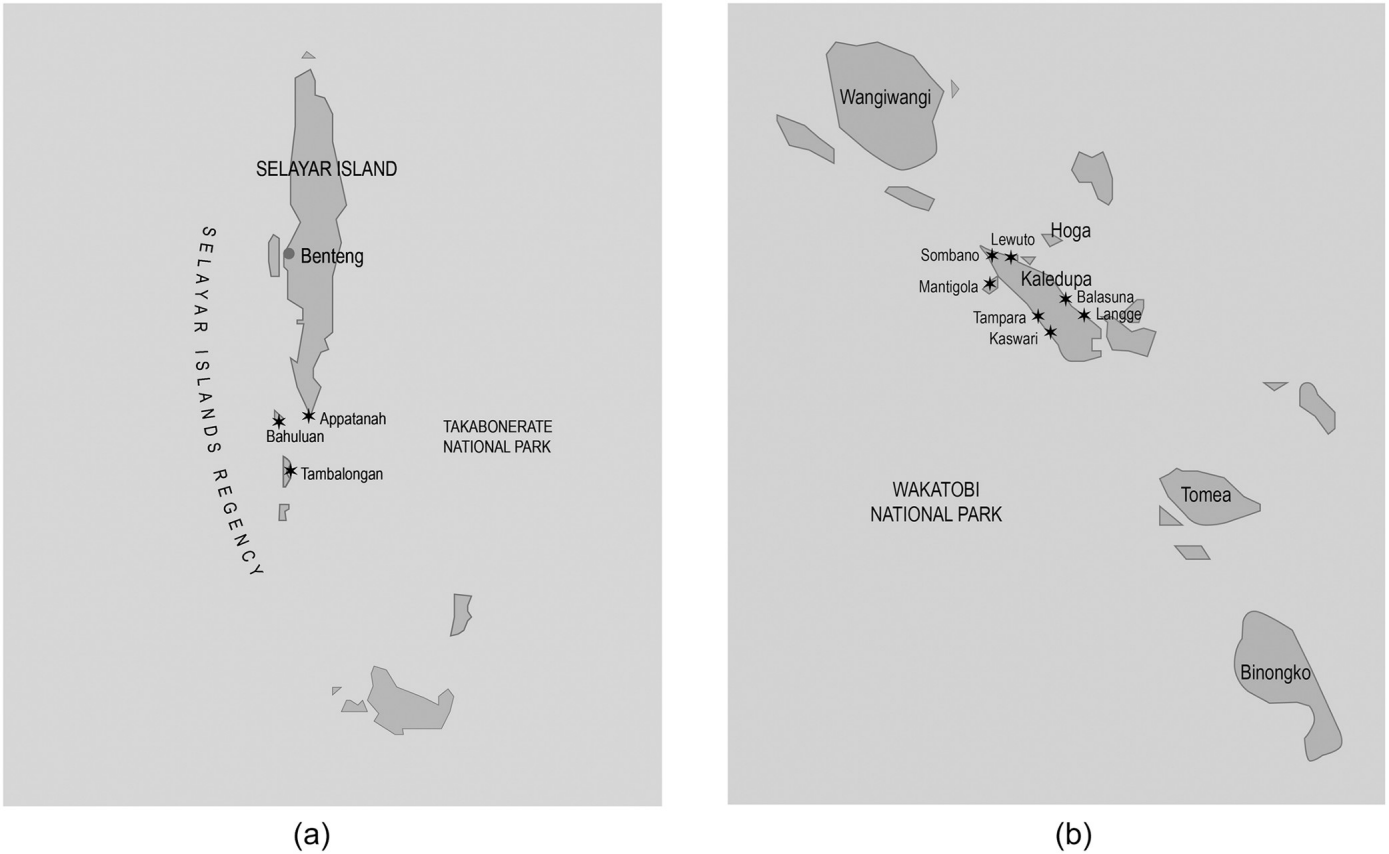


The study was conducted in two coastal zones in southern Sulawesi (Fig 1); first, Selayar, a coastal regency, under the administration of South Sulawesi province, and second, Wakatobi, a coastal regency under the administration of Southeast Sulawesi province, (Fig 2A and 2B) (in the Indonesian system of government, a regency is the next level of administration below a province).

Both regencies are situated within the tropical marine waters of the Coral Triangle, a 6 million km^2^ marine area located in the western Pacific Ocean which encompasses waters of Indonesia, Malaysia, the Philippines, Papua New Guinea, Timor Leste and Solomon Islands (Fig 1). The Coral Triangle is considered to be the world’s epicentre for marine biodiversity, supporting more than 600 of the world’s coral reef species [58], including the highest diversity of reef fish, seagrass, and mangroves [59]. The rich marine life in the Coral Triangle also supports the livelihoods of more than 120 million people and provides resources for millions more [60]. Unfortunately, many marine ecosystems in the Coral Triangle, and in the waters surrounding South Sulawesi, are under threat from anthropogenic impacts including coastal development, pollution, illegal fishing, over-exploitation and climate change [53].

Both Selayar and Wakatobi are in highly biodiverse marine sensitive areas and are part of Indonesia’s marine national park network. They are relatively remote from Indonesia’s main island, Java, and distant from their respective regional centres with reliance on slow boat and air travel: Selayar is 173 km from Makassar, and Wakatobi is 273 km from Kendari and 520 km from Makassar. The nearest recycling facility is in Makassar. The majority of the population at both locations lives on the coast and depends on coastal ecosystems and small-scale fisheries for food and income. Most fishers are artisanal (traditional), with small boats without engines, or with small outboard motors. Most small-scale fishers use hand-lines; other common fishing gear includes spear-guns, raft lifts, gillnets and fish traps [61, 62]. Both sites are prospective ecotourism destinations, and have similar characteristics with respect to waste generation patterns and lack of disposal options.

#### Selayar—Western South Sulawesi

The Selayar islands, situated in south-western part of South Sulawesi, are an archipelago of 130 islands, 26 of which are inhabited. The total area of Selayar Regency is 10,505 km^2^, which includes 1,357 km^2^ of land and 9,145 km^2^ of ocean. The population is 134,280 people with 33,713 households [63]. There are 88 villages in Selayar [63].

Selayar has two seasons. The dry season includes the east monsoon from July to November; and the rainy season includes the west monsoon from January to April. The monsoon periods are particularly significant. During the monsoon periods large quantities of marine plastic debris, carried by ocean currents, arrive from other regions. Selayar’s population comprises the Selayar, Bugis, Buton and Bajo ethnic groups, all of whom live primarily along the coast [64].

#### Wakatobi—Southeast Sulawesi

The Wakatobi islands are an archipelago situated in south-eastern part of Sulawesi. There are four main islands; Wangi—Wangi, Kaledupa, Tomia and Binongko The total area of Wakatobi Regency is 19,200 km^2^, which includes 823 km^2^ of land and 18,377 km^2^ of ocean. The population is 103,450 people with 27,631 households [65]. There are 90 coastal villages in Wakatobi. The population consists of four main ethnic groups; Wakatobi, Bugis, Buton and Bajo, who utilize the marine and coastal resources as their main source of income (fish, seaweed and other marine products). The main indigenous ethnic group, the Wakatobi, are known as *tukang besi* which means blacksmith. The Bajo tribes are known as the former seafaring nomads or sea–gypsies. The Bajo typically construct their houses over water and are particularly dependent on marine resources for their food, shelter, livelihoods, and cultural needs [66–68].

Wakatobi also has two seasons—the dry season, which lasts from April to August and includes the east monsoon (June—September); and the rainy season, which lasts from September to April and includes the west monsoon (December-March).

## Methods

The study used mixed quantitative and qualitative methods [69, 70], in which a survey offered statistically valid representation of the extent of knowledge about plastics and particular behaviours, and ability to relate these to demographic variables; semi-structured interviews provided more detailed information on household waste generation and disposal to expand upon that collected in the survey; and focus group discussions (facilitated with use of a system dynamics app) allowed detailed elicitation and exploration of villagers’ mental models [71] about the place of plastics in the villagers’ lifestyles and livelihoods. The rationale for this combination of methods aligns with Bryman’s categories of ‘completeness’ (that mixed methods allow a more comprehensive account of the area of enquiry) and ‘process’ (that quantitative methods provide an account of structures in social life whereas the qualitative component expands a sense of process).

Ethics approval was granted by The University of Queensland Business, Economics, and Law’s Low and Negligible Risk Human Ethics Sub-Committee (Approval no. 2019000334). The Australian team members who conducted the fieldwork (the first and second authors) hold Indonesian Foreign Research permits granted specifically for this study.

### Village selection

For logistical reasons, we sampled sets of villages that could be reached from a single accommodation base in each study region. The researchers, assistants and survey enumerators then travelled to other villages by boat and vehicle. In Selayar we thus chose: Bahuluang and Tambolongan islands, and Appatanah village on the southern tip of Selayar main island. In Wakatobi we chose Kaledupa island. All selected sites are representative of typical coastal villages in the region which have minimal tourism.

In Selayar, data was collected in the Bontosikuyu Sub-District in Appatanah village on the southern tip of Selayar main island, and Bahuluang and Tambolongan islands. Population statistics are shown in Table 1. There are 12 coastal villages in this sub-district.

**Table 1:** Villages studied—Selayar.

		Total Population	Male	Female	Total Households
	Bontosikuyu sub-district	15,170	7,381	7,789	3,848
No.	Villages				
1.	Bahuluan	318	157	161	84
2.	Tambalongan	1328	654	674	358
3.	Appatanah	870	442	428	207

* Statistical Data of Department of Marine and Coastal Resources, 2018.

In Wakatobi, data was collected on Kaledupa island in seven villages (Table 2). There are 15 coastal villages in this sub-district.

**Table 2:** Villages studied—Wakatobi.

		Total Population	Male	Female	Total Households
	Kaledupa sub-district	10,964			2,847
No.	Villages				
1.	Sombano	930	500	430	170
2.	Balasuna	930	480	450	260
3.	Mantigola	935	465	470	350
4.	Lewuto	640	350	290	180
5.	Langge	1150	545	605	288
6.	Tampara	1076	542	534	244
7.	Kaswari	681	336	345	157

* Statistical Data of Department for Marine and Coastal Resources, 2018.

### Household survey

The survey assessed demographic variables, householders’ levels of knowledge and understanding about plastic waste and ocean plastic, as well as community and personal waste disposal behaviour.

#### Sampling

Owing to lack of a reliable address lists to use as sampling frame, we identified houses on location and through Google© maps. We used a systematic random sampling approach with the aim of sampling one third of the village households. Each enumerator was given a random number between 1 and 3 as their starting point, and given a logical starting point for each day, usually at the end of a beach or street (Selayar, Wakatobi) or agreed starting point on a block (Wakatobi only) (i.e., they would start from the first, second or third house depending on their random number). Enumerators then approached every 3^rd^ house after their start point. In the case of a refusal or no one home, they proceeded to the next house in their sequence until the village or settlement was complete, or the team had to end data collection for that day so that the boat could depart before low tide.

The final sample for the survey was 473, consisting of 210 men (44%) and 263 women (56%). Ages of the participants range from 18 to 78+, with varied levels of education, 57% completing middle school or below. The majority of the sample were long-term residents of the region (15+ years), and were primarily fishers and/or farmers. The sample characteristics are shown in Table 3.

**Table 3:** Sample characteristics.

	n	% of sample
Total Sample	473	-
Female	263	56%
Male	210	44%
Education–Primary and Middle School	269	57%
Education–High School	134	28%
Education–University	70	15%
Long term residents of the region (+15 years)	421	89%
Fishers and/or farmers	194	41%
Housewife	132	28%

Average household weekly income: <US$28.

Average number of people per household: 4.5.

Average age: 41.2.

#### Questionnaire

The questionnaire comprised 38 questions that assessed beliefs, knowledge, and behaviour in relation to plastic waste and waste management more generally as well as demographic variables (Note that only the knowledge, behaviour and demographic questions are reported on in this paper. Please see S1 and S2 Data for the full questionnaire). Fourteen of the knowledge questions formed a plastic knowledge scale, see Table 4 in Results section. The knowledge questions were coded 0 for an incorrect answer and 1 for a correct answer. There were three questions where scores for the questions could be greater than 1 because more than one of the options was correct. The questionnaire was pilot tested during pre-project scoping visits to three remote parts of Eastern Indonesia, including Wakatobi (in different villages to the full survey).

**Table 4:** Individual knowledge scale questions with percent of correct responses.

Single response questions	Correct answer(s)	N	% correct		
1. Organic waste (e.g. food waste, plant litter) thrown on the ground will quickly break down and disappear (become part of the soil).	Agree	473	335 (**70.8%**)		
2. Snack food wrappers and other plastic packaging thrown on the ground will quickly break down or disappear.	Disagree	472	300 (**63.6%**)		
3. The closest recycling facility (plastic processing factory) is located in?	Makassar	473	21 (**4.4%**)		
4. Do fish and other marine animals eat plastic waste?	Yes	471	55 (**11.7%**)		
5. What effect does plastic waste have on the environment?	Negative effect	471	273 (**58.0%)**		
6. Does burning rubbish, including plastic, affect human health?	Yes	471	350 (**74.3%)**		
7. Rubbish left on the ground will eventually make its way into the ocean.	Agree	470	223 (**47.4%**)		
8. In the ocean, how long does the plastic that makes up a plastic bag last?	Up to 20 years	469	143 (**30.5%)**		
9. In the ocean, how long does the plastic that makes up a plastic bottle last?	UP to 400 years	469	66 (**14.1%**)		
10. In the ocean, how long does discarded fishing line does last?	Up to 600 years	467	59 (**12.6%**)		
11. Have you heard about ‘microplastics’—tiny pieces of plastic floating in the ocean?	Yes	470	51 (**10.9%**)		
Multi-response questions		N	1 correct	2 correct	3 correct
12. Which of the following items is ok to throw (discard) on the ground?	Food waste, waste paper	473	238 (**50.3%**)	79 (**16.7%)**	NA
13. Which of the following items can be re-processed in a factory?	Plastic water bottle/sealed cup, coca-cola can, glass bottle	473	223 (**47.1%**)	94 (**19.9%**)	87 (**18.4%**)
14. Do any of the following types of rubbish affect marine life?	Plastic litter, cigarette butts, discarded fishing line	473	315 (**66.6%**)	42 (**8.9%**)	6 (**1.3%**)

#### Procedure

Experienced teams of enumerators, who had worked on previous surveys for other organisations, were recruited. Training was four hours in Selayar, three hours in Wakatobi. Daily team meetings were conducted to clarify issues arising from each day, and plan the following day’s sampling strategy.

The survey was conducted face-to-face in Indonesian language at the respondents’ houses, at a location of their choice (e.g. front verandah). One willing adult from each house was interviewed. The survey was preceded by a short explanation of the study, and ethical points including that participation was voluntary. When respondents did not understand a concept in Indonesian, the enumerators restated it in the local language.

#### Analysis

Analyses were conducted using SPSS [72]. Univariate comparisons (t-tests for comparisons of two groups such as males and females and one-way analysis of variance for other demographic variables involving three or more groups) were conducted to assess any demographic differences on the knowledge scores. In addition, a linear regression analysis was conducted to assess which demographic variables emerged as significant predictors of knowledge controlling for all other variables in the model.

### Semi-structured household interviews

To gauge the amount of household waste produced daily by households of a variety of sizes, and to discuss waste disposal practices in greater detail than was possible in the survey, short semi-structured interviews of 20–30 minutes were conducted at 11 households in Selayar and 14 households in Wakatobi. These used convenience sampling, approaching people at home, aiming for a range of household sizes and prosperity. None of these households were in the survey sample. The household sizes ranged from one to 10 people. The participants were asked about where and how often they shop and what they buy, what they spend, how much waste they generate each day (usually showing us a container), where they dispose of it, and how often. During the interviews we were shown waste piles and household burning sites, and were sometimes taken indoors to see waste receptacles. We were also able to observe household behaviour, such as children requesting pocket money or returning from the kiosk with sweet drinks and packaged snacks. Interview notes and photographs were used to identify common themes and practices. Scales were used to weigh household waste, when possible.

### Focus group discussions using system dynamics application SESAMME

Systems thinking helps to understand and evaluate the utility of decisions made by value chain actors [29, 30]. It provides a systematic framework for articulating, understanding and addressing a ‘dynamic’ problem such as ocean plastic pollution. Specifically, systems thinking characterizes the system structure as a set of interconnected variables and feedback loops, and explains why the existing problem occurs. In this study, we focus on the conceptual component of systems thinking [29], through the lens of community members’ mental models of the causes and effects of ocean plastics.

To explore community members’ mental models [73] of the causes and effects of ocean plastics, and potential solutions, modified focus group discussions (FGDs) were conducted in all of the villages studied. In accordance with standard focus group procedures, group sizes were small and homogeneous, under 12 people, and were moderated to produce an interactive discussion in which participants built on one another’s’ contributions [74]. Unlike the standard, solely verbal, focus group procedure, we used the customised software SESAMME [75] to record the perceived causes and effects of ocean plastics ‘live’ during the discussion as causal loop diagrams. The moderator was a team member and co-developer of this methodology, and a computer-literate local assistant was hired and trained at each location to record the discussion using SESAMME [75].

#### Sampling

Participants were selected and invited by local advisors, following selection criteria based on age (>18), occupation and length of residence (> 5 years). Occupations included: fishers, fish traders, farmers, kiosk or local shop owners, housewives and local government employees. Eleven Focus group discussions were conducted, five in Selayar (one male and one female on Bahuluang and Tambolongan Islands and a joint one in Appatanah village), and six in Wakatobi (one male and one female in each of Tampara, Mantigola and Sombano Villages). The groups were separated by gender, where possible, to allow for the possibility of men and women having different mental models, and to compensate for the possibility of women being less likely to speak out in a mixed gendered group. A summary of the focus group participants is provided in Table 5.

**Table 5:** Summary of focus group participants and summary statistics.

No	Location	Group	No. of participants
1	Bahuluang1	female	12
2	Bahuluang2	male	10
3	Tambolongan1	female	12
4	Tambolongan2	male	12
5	Appatanah–mixed	female	5
		male	7
6	Tampara1	female	12
7	Tampara2	male	10
8	Sombano1	female	10
9	Sombano2	male	10
10	Mantigola1	female	12
11	Mantigola2	male	10
	**Total**		**122**
	female		63
	male		59
	**Summary statistics**		
	Gender:		
	Female (n = 63): 54.64%		
	Male (n = 59): 48.36%		
	Age:		
	Range: 21–62; Mean = 35		
	Education: >50% finished primary school		
	Occupations:		
	Female: Over 75% housewives; others were local government officers, fishers, kiosk owners, or fish traders.		
	Male: Over 50% fishers; others were local government officers, farmers, kiosk owners or small business owners		

#### Procedure

The Focus group discussions were held in community halls and village offices. (Location requirements included: a wall or screen to display SESAMME [75] output, and electricity to run the computer and projector). The discussion topic was “plastic waste in the ocean”. Firstly, participants were asked to identify the activities that influence the problem, and the resources’ which are directly affected by the activities, beginning a causal loop diagram (CLD), please see the analysis sub-section below. Participants were then asked to identify the past, the expected, and the desired trends of each of the activities and resources. Next, the pressures influencing the trends of the activities and the resources were identified and added to the CLD. Afterwards, the participants identified the interaction of each element by mapping direct interactions between resources, activities and pressures using an arrow icon from the apps. Lastly, participants were asked to identify actions which could be taken to address the problem. Each action, similarly, was added to the diagram, including its interactions. The screen depicting the CLD was projected on the wall for the participants to see and follow the process.

#### Analysis

The statistics menu of the SESAMME [75] apps helps to identify common interactions and structure of the mental models across the groups. From this a combined CLD, incorporating those already produced on-screen during the focus groups, can be generated using Vensim^®^ software (developed by Ventana Sytems Inc.). A CLD is a map of a system showing the causal relationships between connected variables [29, 76]. It represents causal relationships between variables that lead to feedback loops that determine the behaviour of the system. A variable is a ‘condition, situation, action or decision’ that can influence, and/or be influenced by another variable [76]. Variables are connected by directed arcs or arrows, which indicate the cause-to-effect relationship. Polarities (plus or minus) are then used to characterize the nature of the relationship between any two connected variables. The polarity is defined as positive (+) if the connected variables move in the same direction; i.e. as the cause variable increases (or decreases), the effect variable also increases (or decreases). The polarity is defined as negative (-) if the connected variables move in opposite directions; as the cause variable increases (or decreases), the effect variable also decreases (or increases).

CLDs, by definition, are focused on representing feedback loops within the system structure, that reveal the dynamic behaviour of a system [76]. A feedback is comprised of a chain of cause-effect connections that connects back to the initial ‘cause’ variable in the chain. In systems modelling, there are two types of feedback loops. Reinforcing loops are positive feedback systems that ‘reinforce’ or amplify change in a system over time. Balancing loops counteract change in a system over time; they seek stability or return to control [76]. Male and female groups were analysed separately, but since there were minimal differences, the results were merged. To simplify output where variables refer to similar activity, resources or pressures were combined as one common variable.

## Results

### ‘Plastic literacy’—Understanding of environmental impacts and recycling

The mean of the knowledge items was used to measure plastic knowledge and the scale had adequate reliability (α = 0.63). The mean score was *M* = 7.12 (*SD* = 3.01) with scores ranging from 0 to 16, suggesting a low level of plastic knowledge overall. Inspection of responses to the individual questions comprising the knowledge scale (see Table 4) shows the lowest levels of knowledge on the following questions: only 4.4% of respondents knew the location of the closest recycling facility, 88.3% did not think that fish and other marine animals eat marine plastic, 89.1%, were also not familiar with the term ‘microplastics’, and a majority (ranging from 69.5% to 87.4%) did not know how long the plastic in plastic bags, plastic bottles and fishing line last in the ocean. Moreover, just over half of respondents (52.6%) did not believe that rubbish left on the ground would eventually make its way into the ocean. On the other hand, a majority (63.6%) of respondents did understand that plastic packaging does not break down when thrown on the ground, and recognised that plastic has a negative effect on the environment (58%), and that burning plastic rubbish can affect human health (74.3%). Responses to the open-ended questions in the survey suggested that respondents perceive plastic waste to be inert or not threatening and that those who perceive plastic to have a negative effect on the environment stipulated that plastic waste has a negative effect on the environment primarily for aesthetic reasons *(‘because it makes the village look dirty’*).

The univariate demographic comparisons showed that householders from Wakatobi had higher knowledge (*M* = 7.32, *SD* = 2.24) than those in Selayar (*M* = 6.47, *SD* = 3.29) (*t*(471) = -3.22, *p* = 0.001), and males (*M* = 7.47, *SD* = 3.07) had higher knowledge than females (*M* = 6.81, *SD* = 2.92) (*t*(471) = 2.38, *p* = 0.018). Knowledge levels were also significantly different between each level of education, (*F*(2, 470) = 65.21, *p*<0.001). Posthoc comparisons showed that those with university education had significantly higher knowledge (*M* = 9.40, *SD* = 2.50) than those with middle or high school education (*M* = 7.66, *SD* = 2.79) and they were significantly higher than those with primary education (*M* = 5.41, *SD* = 2.55). We also explored whether villages that differed in their culture varied in knowledge levels. For example, it may be that Bajo villages—those populated by seafaring nomadic cultures where the researchers observed more plastic and rubbish overall—may exhibit lower levels of knowledge. A comparison of Bajo (i.e., Appatanah, Somabano and Mantigola) versus non-Bajo villages, however, did not reveal significant differences in knowledge levels across culture, (*t*(471) = 1.26, *p* = 0.209; Bajo villages: *M* = 6.81, *SD* = 3.02; non-Bajo: *M* = 7.21, *SD* = 3.00). Knowledge did not differ depending on the income level of the households, (*t*(427) = 0.063, *p* = 0.960; households earning less than 300,000 Rupee *M* = 6.99, *SD* = 2.98; households earning more than 300,000 Rupee: *M* = 6.97, *SD* = 3.08). Finally, age was significantly negatively correlated with knowledge, (*r*(472) = -.319, *p*<0.001).

The linear regression showed that only gender, age, and education emerged as significant predictors of plastics knowledge when controlling for all other demographic variables. Consistent with the results reported above, males had greater knowledge than females (*β* = -0.117, *p* = 0.004), and younger respondents were more knowledgeable than older respondents (*β* = -0.212, *p* < 0.001), and those with higher education (*β* = 0.388, *p* <0.001) had greater knowledge. Neither the site of the village, culture (i.e,. Bajo vs non-Bajo villages) or income emerged as significant predictors (*β*s<0.05, *ps*>0.336). Overall the demographic variables explained 26% of the variance in knowledge, (*R* = 0.513, *F*(6, 461) = 27.51 *p*<0.001).

### Purchasing and disposal

Household interviews and observations showed that typical householders purchase staples (such as coffee, rice, oil, sugar, power milk, etc.), snacks, and other household goods at local kiosks and small village shops daily. Most shops do not have refrigeration. The majority of products come in small ‘sachet’ size single-use plastic packaging and are typically processed or instant food items. Fish is either caught or purchased at the local market. Fishers prefer to sell good quality fish to fish collectors and keep smaller lesser quality fish for their own consumption. Very few vegetables are grown on the islands due to poor soil quality. Vegetables are brought in by boat and sold at the local markets. Chickens and goats are common in villages and help to dispose of kitchen waste. Many households reported that processed packaged foods from stores were cheaper and more convenient than fresh foods, and so were preferred. The most popular items are processed snacks which are primarily purchased by children with pocket money that their parents readily provide, despite low incomes. Some of the respondents with boats would travel long distances, and frequently, to shop at cheaper stores, finding the savings outweighed fuel costs and inconvenience. Most, however, preferred daily local shopping.

During the 25 semi-structured household interviews, household waste volumes were estimated through weighing household rubbish, observation, and discussion. Average daily household waste was estimated at 2 kg/day or 14 kg/week. Typical daily household waste included: kitchen waste, garden leaf litter, cardboard packaging, plastic bottles and packaging, cigarette packaging, glass bottles, paper, and miscellaneous items. Waste from recreational activities (such as picnics and sporting events) and community gatherings (such as weddings and parties), was estimated at .25/kg of plastic waste per person per event (based on observation, discussions with informants, and by weighing resulting plastic bottles and packaging from observed community events). This number was multiplied by the average household size, which the survey indicated was 4.5, resulting in an estimated total of 1.125 kg/household/week. Fishing and boat travel was estimated in the same way based on the assumption a typical household will do at least one inter-island round trip per week (resulting in .25/kg of plastic waste per person or 1.125 kg/household/week). Estimated household waste flows are presented in Table 6.

**Table 6:** Estimated household level waste flows per week.

	kg per week
Average waste from a typical household (4–5 people) based on 2kg/day per household	14
Waste from recreational activities produced by members of household	1.125
Waste from community gatherings, weddings and other special events; Aggregated per household	1.125
Fishing and boat travel–estimated per household	1.125
**Total household waste**	**17.375 kg/household/week**

Multiplying the estimated total household waste (17.375 kg/household/week), and the mean number of households in the villages studied, 230, we estimate that at the household-level a typical village generates approximately 4,000 kg of rubbish per week including plastic. In our survey nearly half (48%) of respondents stated that in their community most of the rubbish is burned, and 25% stated that most of the rubbish is thrown in the ocean. Applying the assumption that the rubbish that is not burned makes its way into the ocean, we estimate that approximately half of the rubbish produced by a typical coastal village, or 2,000 kg/week, leaks into the ocean. This result is relevant because there are tens of thousands of typical coastal villages in the Indonesian archipelago.

None of the villages that we focused on in our research have regular waste collection services or a ‘garbage bank’. A garbage bank is a term used in Indonesia for a facility where empty plastic containers can be sold and are sorted, shredded and moved down the value chain. Interviews and observations revealed that households dispose of their rubbish, including plastic, either by burning in small frequent bonfires in the yard, throwing it directly into the ocean (often in a plastic bag), or piling it behind the house. Some is piled up and then burnt. Mangrove areas and other wetlands are also used for rubbish dumping and disposal. Rubbish is also used to fill-in mangrove areas as part of a process for converting wetlands to dry land. In other communities observed in our scoping visits, rubbish is also dumped in drains and streams, later to be carried to the ocean.

Survey results showed that convenience is important, with 92% of respondents confirming that they are only prepared to walk very short distances from their house to dispose of their daily rubbish. Observations and interviews showed that plastic waste from snack products is typically dropped on the ground, in the street or yard. If dropped in the yard, it is swept up every one to two days and then disposed of as above. Most households burn their household waste, including plastic and leaf litter, once or twice a week. Some households burn rubbish every day. Some householders report taking care about timing, avoiding winds and breezes.

Disposable diapers or nappies are widely used, a relatively recent phenomenon both in Selayar and Wakatobi. Kiosks and local stores sell nappies packaged individually. Parents interviewed reported using 2–3 nappies per day. Nappies are disposed of directly in the ocean, occasionally they are dumped with other rubbish. It is considered taboo to burn human waste; as a result most used nappies make their way into the ocean.

In the survey, only 30% of respondents said that plastic waste (bottles, wrappers and other packaging) should be put in a bin. Instead, 59% said it should be burned, 3.8% said it should be buried, and 13.5% said it should be put in the ocean. Although the majority of survey respondents (74.3%) said that burning plastic affects human health, especially as it *‘causes coughing and makes it difficult to breathe’*, interviews and focus group discussions confirmed that most community members will continue to burn plastic as there are no other options for disposal. Household interviews revealed that plastic waste is not prioritised in the community or household clean-ups, because, unlike organic waste, it does not have a smell and so is considered benign. The interviews revealed that the smell from decaying organic rubbish is often associated with ill-health. At the time of the study, bins for street collection had just been introduced in two Kaledupa (Wakatobi) villages, but collections were not well established and households were not accustomed to using them yet. There was no managed landfill for disposal of the wastes once collected. Open burning at dump sites was observed frequently.

### Mental models of the plastics ‘system’

Focus group discussions using SESAMME allowed community members to build collective mental models of the causes and consequences of ocean plastics. Combined results are presented here.

#### Activities contributing to plastic in the ocean

Participants identified seven activities that contribute to the amount of plastic waste in the ocean (boat travel, dumping, fishing, kiosks, seasonal monsoons, community gatherings, seaweed farming), shown in Table 7, and one that ameliorates it (e.g. beach cleaning).

**Table 7:** Village activities contributing to plastic in the ocean.

*Boat travel* (10 groups)	Participants explained that as the communities live on small islands, the need to commute between islands is unavoidable. Increasing household income, which makes travelling affordable and increases the number of children able to attend high school on the main island, and increasing population, are among factors affecting the frequency of boat travel. Many passengers bring food and drinks for the trip and dump their plastic packaging into the ocean.
*Dumping household rubbish directly into the ocean* (10 groups)	This is commonly practised by many members of coastal communities. The amount of plastic wastes continues to increase as the population increases. Participants also mentioned that rubbish brought in by the currents made them less hesitant to dump their household rubbish in the ocean.
*Fishing activities* (7 groups)	Discarded or lost fishing lines, nets, and other broken fishing gear also contribute to the amount of plastic in the ocean.
*Growth in the number of kiosks* (6 groups)	Population growth creates more demand for snacks and drinks, leading to more kiosks opening.
*Seasonal rubbish washed up from the ocean* (6 groups)	Although this phenomenon occurs mainly in the monsoon months, the amount of the rubbish is overwhelming and many orders of magnitude larger than that produced by the village. Accounts describe it as a ‘landfill dumped into the ocean’. Rubbish remains on the beaches year after year, or is washed back into the water, if not addressed.
*Community gatherings and meetings* (3 groups).	Growing population means more community gatherings such as betrothals, weddings, and picnics, and meetings, at which water is commonly served in single use plastic cups or bottles.
*Seaweed farming* (2 groups in one village).	The people in one village in Wakatobi are mostly seaweed farmers. Seaweed farming uses floats made from fragments of polystyrene and used plastic bottles, which can break away and remain in the ocean.

Out of 11 focus group discussions, only three (all in Selayar) mentioned holding beach cleaning activity to reduce the amount of rubbish left on their beaches.

#### Activities affected by plastic in the ocean

**Fig 3:**
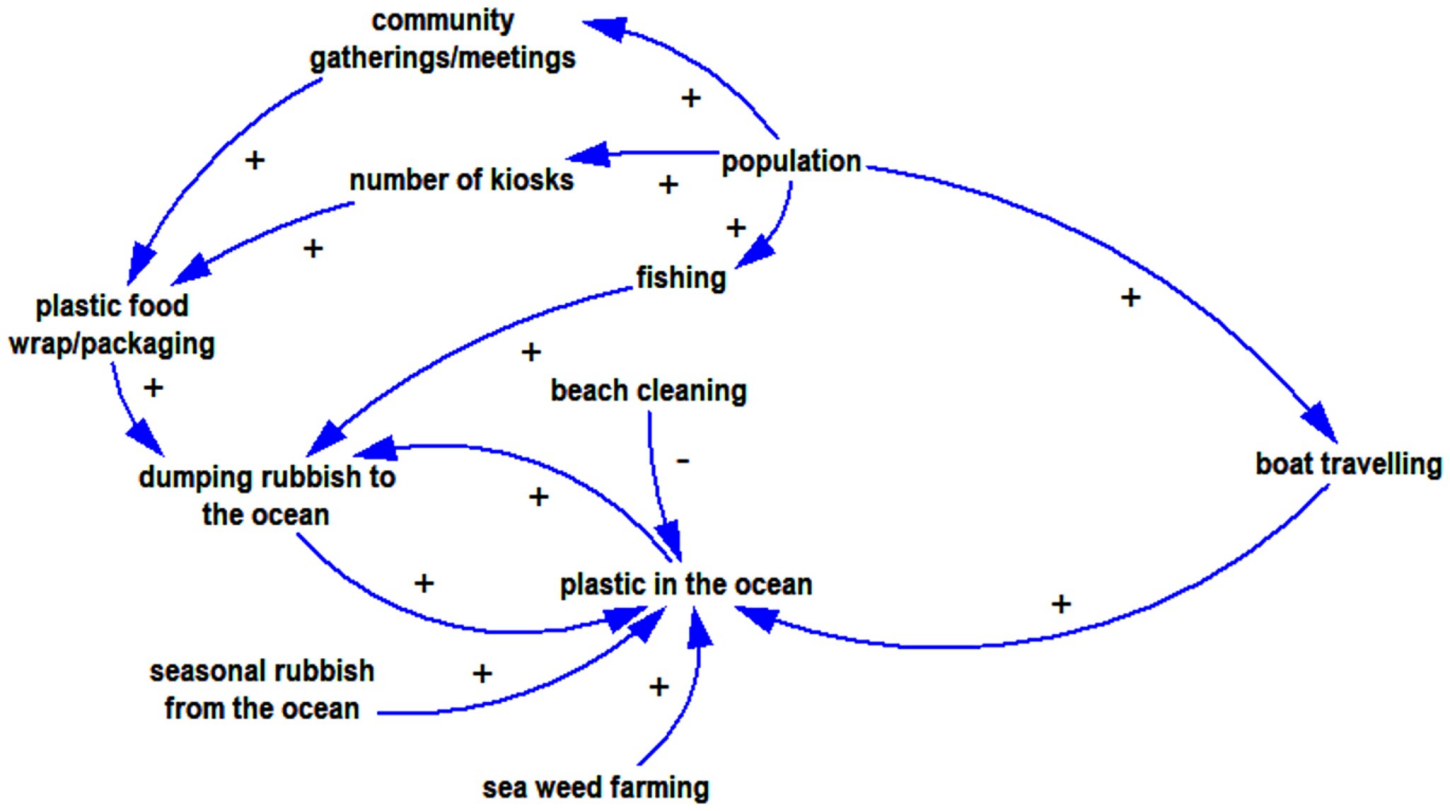


The Focus group discussions revealed two activities directly affected by plastic in the ocean, fishing and seaweed farming. Plastic waste damages fishing gear and propellers, so fishers must waste time in repairs, and this forces them to do more fishing to earn income. Plastic also often interferes with seaweed farming by fouling the floats and lines so that the seaweed is not held at the best depth. Other activities related to fishing affected by ocean plastic include interference with loading and unloading, and landing sites clogged with rubbish, especially during low tide. All the activities contributing to and affected by plastic in the ocean are presented in Fig 3.

#### Resources affected by the plastic in the ocean

The FGD participants identified seven resources as negatively affected by plastic in the ocean: fish (9 groups), the attractiveness of beaches (4 groups), coral reefs (4 groups), sea grass (4 groups), mangroves (3 groups), water quality (2 groups) and seaweed (2 groups).

*Fish and fish habitats*. Participants mentioned that more plastics in the ocean will directly reduce the number of fish in their area and more plastic will damage reefs, seagrass, and reduce water quality. All of these will also reduce the fish population. Once habitat is damaged, fish stock will decrease. Less fish will reduce the fishing yields and reduce incomes.

**Fig 4:**
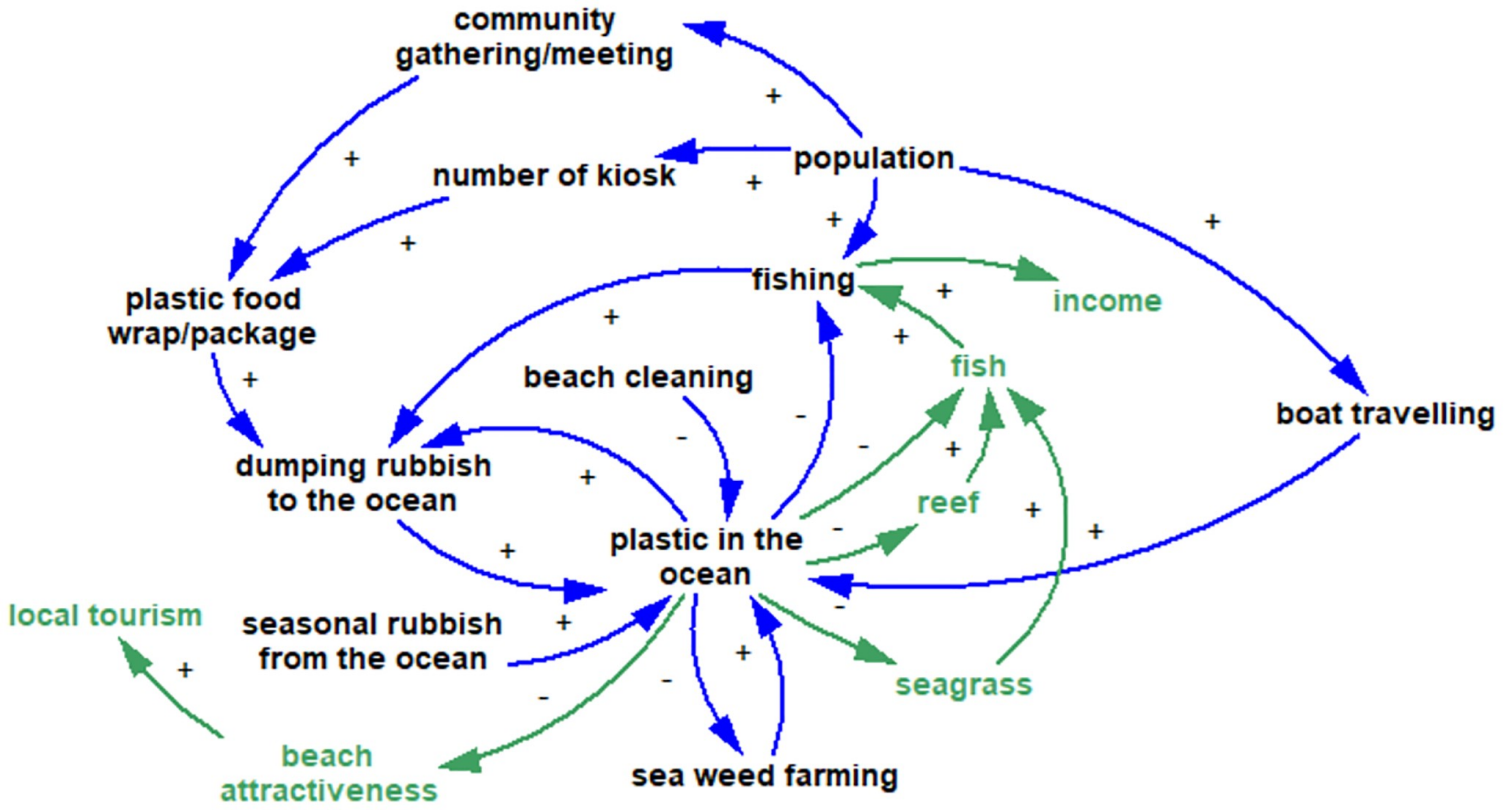


*Beach attractiveness*. Also, more rubbish ruins the appearance of the beach and damages the mangroves which then reduces the tourism potential (Fig 4).

#### Amelioration activities

Participants mentioned that efforts have been made in some villages to reduce the dumping of rubbish into the ocean. These include burning the dry waste (all villages) and providing rubbish bins using village funds (very new, in some Wakatobi villages).

### System archetypes

Archetypes are generic systems structures which describe the common dynamic processes of the system [29, 76]. Finding archetypes helps to identify the leverage points for intervention in the system [30]. System archetypes provide a platform from which to share dynamic insights [77]. Analysis of the model identifies three types of archetypes; ‘limits to growth’, ‘escalation’, and ‘fixes that fail’.

#### Limits to growth

**Fig 5:**
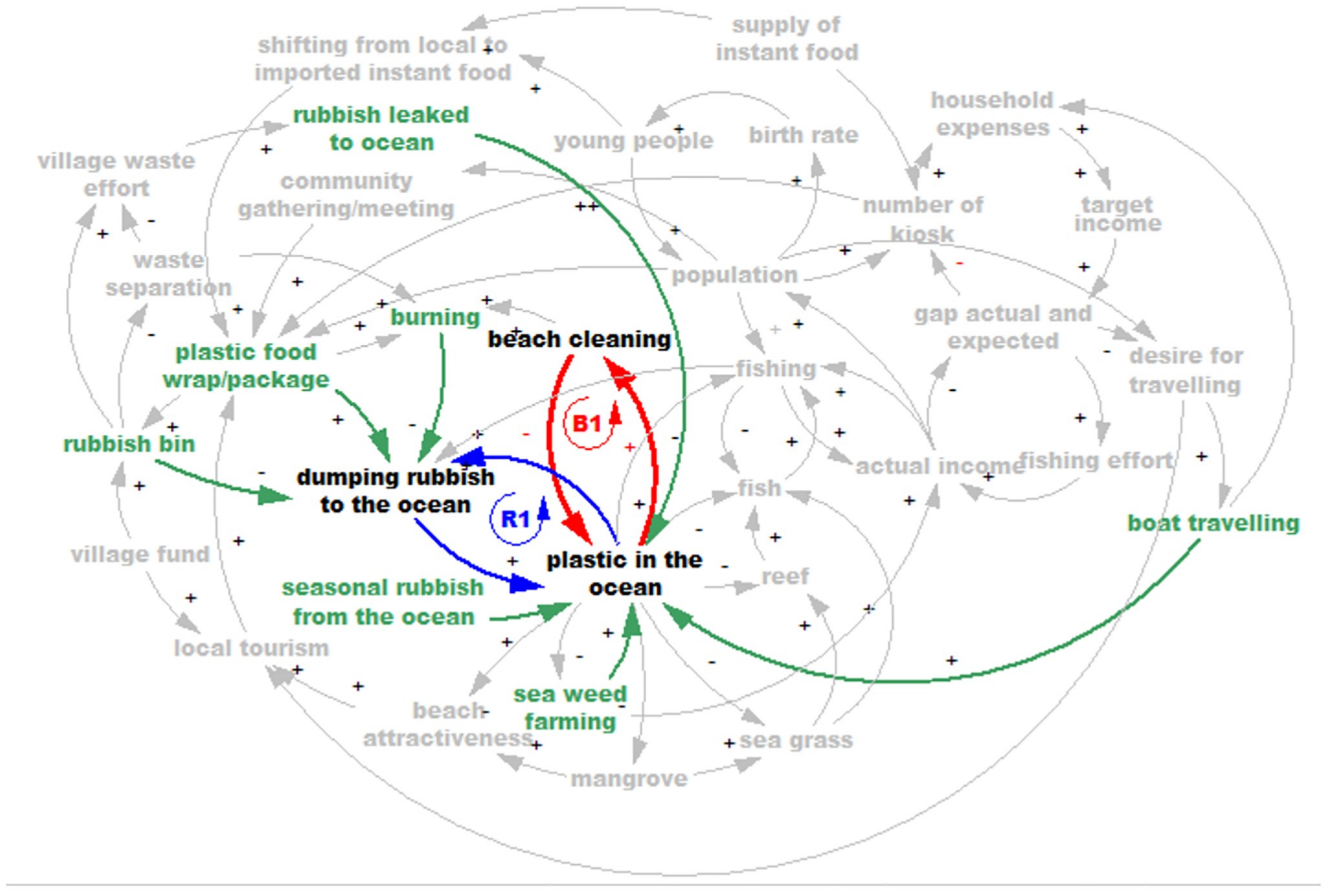


‘Limits to growth’ is one of the most well-known archetypes [78]. It reflects the manner in which initial growth may be slowed over time by a limiting factor [30]. The model structure that produces this dynamic consists of at least one reinforcing loop (positive feedback) and one balancing loop (negative feedback). This archetype describes a process in which a period of accelerating growth is followed by a period of deceleration [30]. The limits to growth as related to plastic is shown in Fig 5, with full model shown in grey.

The reinforcing loop (R1) explains that increasing plastic in ocean means people will throw even more rubbish into the ocean. R1 is followed by a balancing loop (B1), which suggests that beach cleaning efforts will reduce plastic in the ocean. Several important factors affect the rate of the R1 loop: infrequent beach cleaning; seasonal rubbish brought ashore by the ocean currents; the ever-increasing extent of plastic food wraps and plastic bags from groceries; broken Styrofoam floats and plastic bottles used for seaweed farming.

#### Escalation archetype

**Fig 6:**
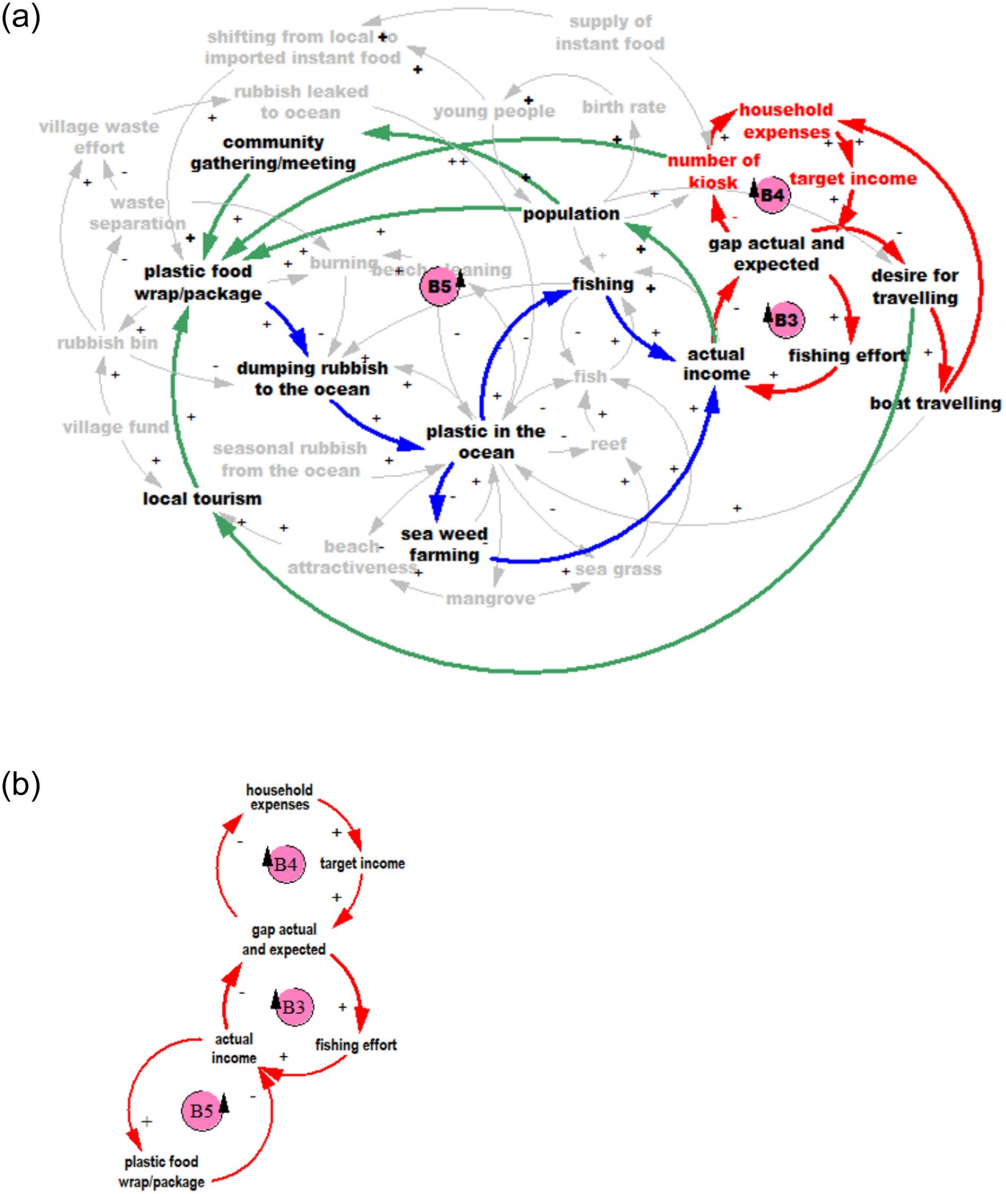


This archetype describes a situation in which when one loop advances, another loop is threatened and acts more aggressively to gain advantage. This in turn threatens the first loop, increasing its aggressiveness, and so on [30]. Fig 6 demonstrates this archetype.

The model Fig 6 shows that plastic in the ocean will keep increasing as a result of an escalation archetype (B3 and B5). More income will enable households to buy more groceries, and do more travelling, which leads to more plastic waste. The archetype indicates that people will try to increase or at least maintain their level of income which results in producing more plastic waste.

#### Fixes that fail archetype

**Fig 7:**
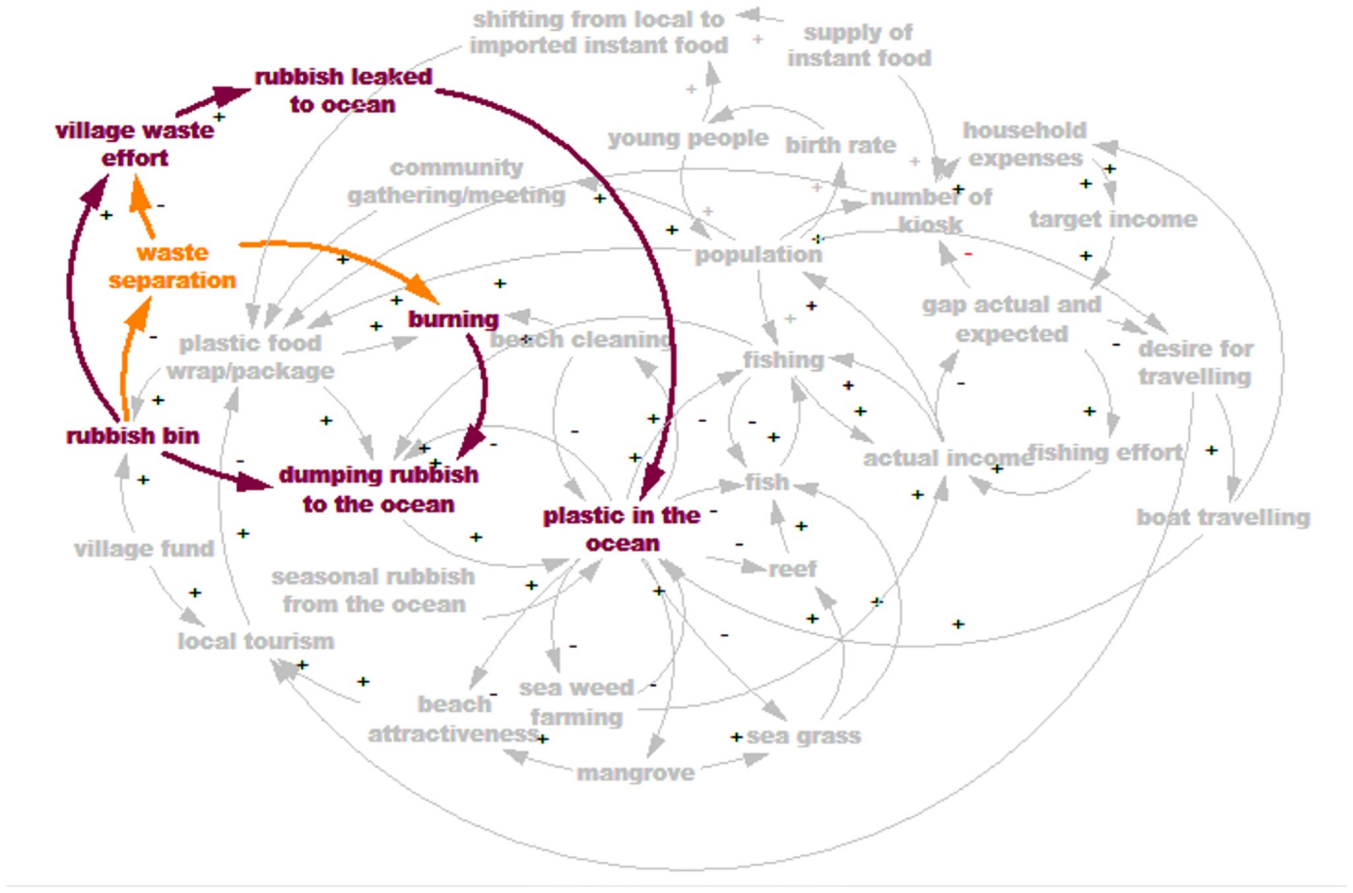


This archetype describes a situation where an intervention seems to be effective in the short term, but has unforeseen consequences in the longer term which may need even more interventions [30]. Fig 7 shows the fixes that fail in the model.

There are two activities currently practised by these communities to manage their waste; (1) burning the “dry” waste (such as, leaves, plastics, paper); and (2) providing rubbish bins at the front of the house (newly introduced, in some Wakatobi villages only). Burning will reduce the amount of rubbish dumped to the ocean. However it has negative effects such as air pollution, irritating eyes, noses and lungs. Providing rubbish bins, with a collection service, should reduce the dumping of rubbish in the ocean, but as there are is no waste treatment or landfill on any of the islands studied, all of the waste collected will be dumped at an un-managed dump site located at some distance from the settlement. Observation showed that some of these disposal areas are close to mangroves, and houses. More rubbish sent to poorly controlled dump sites could result in a higher proportion of the rubbish flowing to the ocean. Thus, providing rubbish bins, which seems a good solution initially, in the long term may not reduce plastic inputs into the ocean.

**Fig 8:**
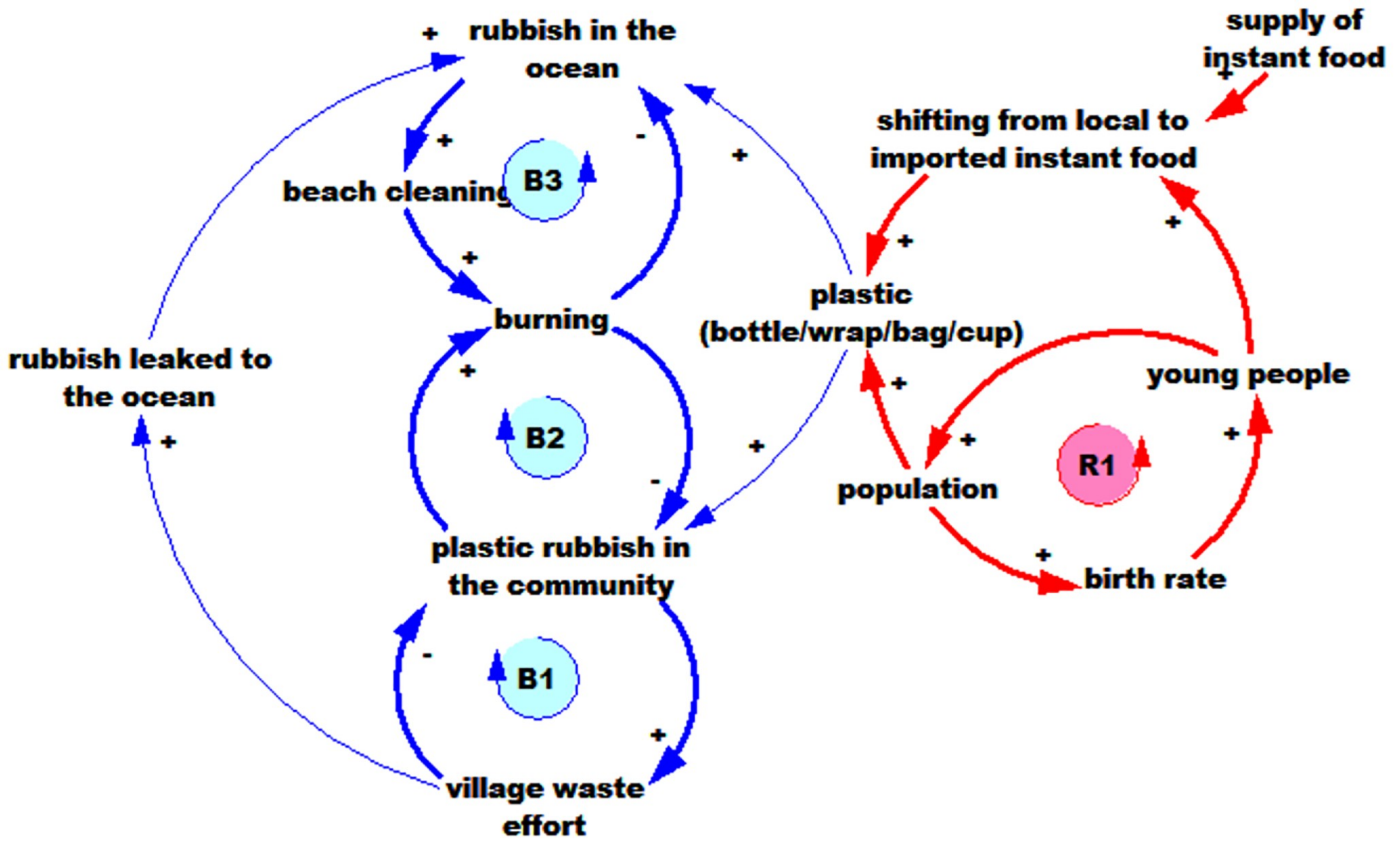


From the shared mental model we are able to identify that although communities suffer from plastic wastes, some of them keep dumping their rubbish in the ocean because they feel that their contribution is insignificant compared to the amount of rubbish already there, visible in the quantities washed up on their beaches annually. Also, the model shows that an obvious intervention such as distributing rubbish bins to the community is not always the best solution (and even sometimes creates more problems) unless attention is paid to more fundamental as aspects of waste management. Fig 8 demonstrates the plastic waste dynamic for both sites.

The shared mental models show that the dynamics of the plastic rubbish in the ocean in both study locations revolve around a combination of factors. One is the rising inflow of packaged goods contributing to plastic waste on the islands, (i.e. the incidental supply chain of plastics associated with foods, drinks and other consumer goods), and the other is how plastic wastes on the islands could be managed so as not to end up in the ocean.

## Discussion

The study aimed to understand the factors that contribute to the use and management of single use plastics in coastal and island communities in Eastern Indonesia, and to offer new insights into unmanaged plastic waste and the support that is required to reduce the problem. This is a critical issue when considering that small remote villages account for a large proportion of the coastline in Indonesia [79], and such communities have far less capacity to address the issues than their urban counterparts. Results from the survey indicate that knowledge about plastic and how to manage it is relatively low. This finding was mirrored in the household interviews and observations, although these latter methods also highlighted the reasons for the increase in plastics in villages and the barriers that exist to appropriate management of plastic waste. Villagers reported that foods that come in plastic packaging are often cheaper and more convenient than fresh foods, and in all the villages that were part of our study, there were no regular waste collection services. This lack of infrastructure meant that villagers have to find alternatives: burning or dumping their waste, sometimes in piles that can be washed away in heavy rains, sometimes directly in the ocean. Our estimates from household interviews suggest that almost half of the rubbish produced by the typical village may ‘leak’ into the ocean. Importantly, the findings from the focus groups allowed us to tap into the mental models of villagers so that we can better understand the causes and consequences of ocean plastics. This method provided deeper understanding of the role of plastics in people’s lifestyles, livelihoods and environment, and also of their awareness, than the other methods did. Villagers identified activities that contribute to ocean plastic including boat travel, weather (seasonal monsoons), fishing, seaweed farming, kiosks, dumping and community gatherings. On the other hand beach cleaning can help to reduce the problem. Villagers also identified that the livelihood activities of fishing and seaweed farming are affected negatively by ocean plastic. In the following sections we discuss these findings in more detail, drawing out the implications for managing plastic waste in these low resource coastal communities.

### What mental models reveal

The conceptual models represented in Figs 3–8 represent shared mental models, built from collective discussion among local men and women in the participating communities. A mental model is a ‘model in the mind’ [73]. They represent local knowledge, and assumptions based on their experience. They can also have gaps or distortions compared with what might be found by other observers or objective measures. For instance, while all interactions here are verifiable and tally with the research team’s observations, factors of knowledge or awareness and individual behaviour change—two favorites in environmental literature—are notably absent. The participants do refer to village-organized waste disposal; this is the level of government most likely to organize waste disposal in small, remote islands. Does this mean the local people see no role for improving awareness and changing individual behaviour, do not feel local households are part of the problem and solution, or that those factors are far outweighed by others? The mental models recognize the behaviour pattern of dumping rubbish in the oceans, and indeed draw out new occasions when it happens (e.g. during boat trips and social gatherings), yet do not include building awareness or behaviour change as interventions that could offer balancing loops. They do refer to beach cleanups, a collective behaviour.

The interactions presented through the closed loop diagrams have the advantage of showing levels of local nuance, insider revelations about how plastics in the ocean relate to the everyday lives of the relatively poor. In doing so they complement the growing literature on physical aspects of the problem (the extent and locations of plastics in the ocean, the need to improve waste management) [1, 4].

While it is a shared mental model based on local people’s understandings of causes and effects of plastic pollution, this matches well to the team’s observations in the field, household interviews, and survey results. It shows a complex system, in which there are multiple causes of the extent of plastics in the ocean, inter-related with factors such as increasing income and population growth. Interestingly, but perhaps not surprisingly (as noted above), community awareness is not mentioned specifically in the mental models, though it is somewhat apparent in the noting of behaviours such as throwing rubbish overboard during boat trips. Shared models also showed that the vast quantity of seasonal rubbish brought ashore in the storm seasons discourages the community from cleaning the beaches, and encourages more disposal of rubbish to the ocean as individuals perceive their additions as insignificant compared to the piles of rubbish brought in by the currents.

Lack of choice in relation to disposal options underlies parts of the model. Food shopping options are biased towards packaged convenience foods, and there is a severe lack of safe and convenient waste disposal options for households and communities. Our observation suggests the latter is not easily resolved. None of the islands appears to have land suitable to turn into landfills, recycling is not easily viable so far from the main Indonesian recycling stations (and does not deal with the many other types of waste), and lack of affordable small-scale technologies so far precludes safe incineration in villages. Even if effective waste management were available, collection services at a household level would also need to be established—survey results and the household interviews suggest that convenience plays an important role in waste disposal behaviour.

We elucidate further on the system archetypes revealed through focus group discussions to help further explain the resulting dynamics. The limits to growth archetype, shown in Fig 5, suggests that the plastic waste in the ocean will keep increasing. Rubbish brought ashore by the ocean currents, broken Styrofoam floats and plastic bottles used for seaweed farming, single used plastics dumped from boats, and rubbish leaked from the unmanaged village disposal, outpaces any waste management and beach cleaning efforts. Furthermore, the extensive accumulation of plastic waste on beaches and coastlines discourages villagers from taking any mitigation action themselves, thus resulting in their continued dumping of rubbish into the ocean, as shown by reinforcing loop, R1, in Fig 5.

The escalation archetype, shown in Fig 6A and 6B, focuses on the household need for greater income (B4). As most of the community members are fishers, we use the example of fishers needing to increase their fishing effort to gain more income (B3). More income allows purchase of more groceries and single-use plastic packaging, thus leading to more plastic waste (particularly in villages where there are no waste management systems). Plastic in the ocean damages fish habitat (reef, mangroves, and sea grass), interferes with sea weed farming, and fishing activities through propeller entanglements and polluted landing sites. These reduce fishers’ incomes. This induces the fishers to increase their fishing effort, and the cycle continues to escalate.

The fixes-that-fail archetype captures the quick fix solution with potential unintended consequences. The mental models reveal that village beach clean ups, although beneficial in ameliorating ocean plastic, have led to increased burning of plastic waste (due to lack of other disposal options). The resulting localised air pollution is creating respiratory concerns such coughing, itchy and burning eyes (shown in the survey results), with potential long term health implications. Similarly, the very recent introduction of bins by some villages has affected traditional disposal practices—separation of dry waste (paper, leaf litter, plastic) and organic kitchen waste (typically left out for chickens and other animals), and has led to overflowing unmanaged village dump sites, which also present a health hazard.

### Driving factors for plastic waste outpacing mitigation efforts

The findings from the focus group discussions, household interviews and observation indicate that daily use of single-use plastics is outpacing standard disposal methods, such as burning and dumping (either directly in the ocean or behind the house). This supports existing literature which states that availability of fast moving consumer goods and the rising standard of living is driving plastic waste accumulation rates in emerging economies [4, 39, 80, 81]. Our findings also confirm that seasonal storms and high transportation costs make waste management a difficult issue for remote Indonesian communities [9, 82]. The accumulation rates of plastic on the coastlines, in some regions, is reaching catastrophic proportions [83, 84]. When plastic is improperly discarded, it will find its way into waterways and the ocean [3, 6, 85]. In most coastal communities waste never makes it to a landfill, or anywhere near a recycling facility. Villages studied for this research did not have effective infrastructure to collect and recycle plastic waste.

In remote coastal communities in Indonesia waste management entirely depends on village funds and provincial government support (and our observation is that this is not a vote-winning issue for political candidates). Waste management infrastructure necessary to keep up with waste generation rates is unattainable for small low-resource communities. Large scale centralised waste management is very challenging due to complex coastal geography, distances between islands and high transportation costs. Furthermore, relationship with the ocean and dependence on the ocean to meet daily needs, including waste disposal, is linked to long standing cultural traditions and beliefs [68]. The plastic literacy survey conducted as part of this study demonstrates that understanding of the environmental impacts of plastic is low. Better understanding of environmental impacts on the part of the community would not necessarily help, however, as effective waste disposal options are limited. Nevertheless, the low plastic literacy suggests that even if adequate waste management was introduced, there would be a need to accompany it with education and awareness raising.

As findings from the focus groups suggest, seasonal monsoons play an important role in the amount of plastic villagers encounter. During the east and west monsoons, the situation reaches extreme proportions. A monsoon season lasts for several months. The high winds and storms bring high volumes of marine plastic debris on ocean currents inundating the coastlines of island communities. Large volumes of marine plastic debris interfere with fishing and other livelihoods. Most communities are not equipped to manage large-scale clean up. As noted in the focus groups, weekly beach clean-ups in a few of the communities, mostly done by hand, are unable to deal with high volumes. This, coupled with lack of disposal options with burning being the only alternative, results in most of the marine plastic debris left on the coastlines. The villages studied left the majority of marine debris on beaches. A handful of tourism and dive operators encountered in the adjacent national parks confided that they struggle to keep up, spending considerable amounts of time, money and effort to stem the plastic tide.

Focus group discussions also showed that the seasonal replenishment of rubbish discourages community members from doing beach cleaning and encourages more rubbish to be thrown on the ground or in the ocean. Meanwhile, growing population [86] and increasing standard of living is creating a demand for single-use packaged goods [82]. Social mobility is incentivising parents to provide store bought snack foods, which were not affordable to them when they were children. The abundant snack foods available at local kiosks such as soft drinks, instant noodles, crisps, biscuits and chocolates, are primarily bought by children with pocket money that their parents provide.

Taking into consideration lack of formal plastic waste management systems, low plastic literacy rates, increased availability of packaged processed food, and the occurrence of seasonal monsoons that bring large volumes of additional plastic marine debris, it is safe to surmise that plastic waste is increasingly outpacing mitigation efforts in remote, coastal communities. In particular, sachet waste and empty plastic beverage containers dominate the coastal regions. Typically, producers and manufacturers do not internalise the costs associated with waste management and environmental impacts.

Globally, the value chains of single-use plastic packaging are still predominately linear, with 95% of the material value of plastic packaging (and estimated US$100bn annually) being lost after a single use [5]. Although prevalent throughout most of the developing world, the sachet economy is underrepresented in efforts to shift to a more circular plastic economy [38, 87, 88]. At the centre of the plastic waste problem is the linear ‘take-make-dispose’ model of consumption [89], which means products get manufactured, bought, used briefly, and then thrown away. For the FMCG sector innovation and new business models are required to ensure that plastic does not end up as waste, or worse, polluting marine and coastal ecosystems [90]. To protect the world’s oceans, rethinking plastic packaging towards a more circular approach—where packaging is designed so that it can be reused, recycled or composted—is a matter of urgency [91], especially for producers supplying to remote, coastal communities.

### Effects on livelihoods and health

Findings from the study demonstrate that ocean plastic affects the daily lives of people living on remote coastal communities in numerous ways. Coastal and island communities directly depend on healthy marine ecosystems for food, livelihoods, income, cultural, recreational, and spiritual needs [23–25]. These communities are particularly vulnerable when marine ecosystem function is reduced or affected [1, 60]. Fishing is the primary livelihood in the region, and fish the primary source of protein [62, 92]. The shared mental models, survey and interviews show that members of the community perceive and have experience of marine plastics having a negative impact on fishing, seaweed farming, and emerging regional industries including tourism.

Survey responses showed that most respondents recognised the negative health implications of burning plastic waste; a common and preferred method of waste disposal. Single-use plastics are a relatively recent phenomenon in remote island communities of Indonesia. Due to minimal disposal options, and perceptual issues about the relative relevance of organic waste and plastics, each village household typically combines plastic waste with leaf litter and other household rubbish into a pile. The rubbish piles are burned once or twice a week. The practice produces toxic fumes and contributes to air pollution [93–95]. The situation is exacerbated during monsoons when large volumes of marine plastic are disposed of through burning by those coastal villages attempting clean-ups.

### Research limitations

The results of this research should be considered in the light that two locations, involving 10 villages, were studied. A scoping study and the team’s other research in other parts of Indonesia suggest that these villages, and the experience of ocean plastics, household consumption and disposal behaviour, and absence of formal waste disposal, are typical for remote non-metropolitan areas. Nevertheless, any generalisation should be approached with caution, particularly outside Indonesia. The survey and focus group results are based on robust sampling, with a sizeable proportion of each village completing the surveys. One issue that should be considered is whether survey responses were influenced by which member of the household responded to the survey. Fifty-six per cent of survey respondents were women, relative to 49.5% of women in the villages studied (see Tables 3 and 4). In the survey, we suspect possible response bias in one behavioural question about where the households dispose of their rubbish: since many households are aware that disposal in the ocean is frowned upon, numbers claiming this method of disposal may be an underestimate. Other questions appear to have been answered honestly (and respondents were ready to say ‘don’t know’ where that applied). The household interviews were sufficient for their purpose, however, convenience sampling and potential imprecision with which some householders’ described their daily and weekly purchases and daily rubbish amounts should be considered. This was somewhat compensated by the opportunity to see these behaviours, and signs of disposal, at first hand.

## Conclusion

The oceans continue to fill with plastic litter, most of it single use plastic and retail packaging. At a systems level, social and economic costs are often borne by those affected rather than those responsible for the supply of the plastics and management of the wastes. This research confirms that for coastal and remote communities in Indonesia, the use of plastics is increasingly overwhelming waste management and infrastructure capacity.

This study showed that low-resource coastal communities in South Sulawesi are forced to shoulder the impacts of the ocean plastic crisis. There are thousands of similar coastal communities in Indonesia, all struggling to cope with their own waste, plus vast quantities of waste brought in by currents. The system results show that communities are caught in a perpetual reinforcing loop. Unless the supply changes, these communities have no hope of effectively managing their waste. Although our survey results show that plastic literacy is low, even if it were higher there is little the coastal communities can do to manage plastic waste effectively unless presented with better choice architecture, both on the supply side and in disposal options. For coastal communities in emerging economies the ocean plastic crisis cannot be abated without responsible supply.

Although the issue of plastic pollution has to be solved on multiple fronts [40], our data suggests that for coastal communities extended producer responsibility is imperative and a circular plastic economy is greatly needed. Coastal communities with minimal waste infrastructure require circular systems and responsible supply chains with non-plastic alternatives. Producers and manufacturers distributing low-cost processed food and single-use products to remote, coastal communities need to take responsibility for the outcomes. Without access to degradable, environmentally friendly products, and a circular plastic system, coastal communities and surrounding marine ecosystems will continue to drown in plastic waste.

## Supporting information

S1 Data Survey questionnaire Bahasa Indonesia. (DOCX)

S2 Data Survey questionnaire English. (DOCX)
