# Supplementary material for: Ocean plastic crisis—Mental models of plastic pollution from remote Indonesian coastal communities
Source: PLoS One. 2020 Jul 28;15(7):e0236149. doi: 10.1371/journal.pone.0236149 (PMC7386615; doi:10.1371/journal.pone.0236149)
Supplement: S1 Data — (DOCX) [file pone.0236149.s001.docx]

**Proyek:** INISIATIF PULAU-PULAU KECIL UNTUK MEWUJUDKAN LAUT BEBAS SAMPAH PLASTIK

***Tanggal: Kode Responden:
(Nama enumerator; responden nomor)***

***Nama desa: Nama dusun (jika ada):***

Lokasi rumah tangga:

|  | - 1. Dekat pantai |
| --- | --- |
|  | - 1. Di tengah desa |
|  | - 1. Di belakang desa |

1. Apakah permasalahan terbesar yang anda hadapi di lingkungan anda?
2. Sampah organik (sampah bekas makanan, sampah yang berasal dari tanaman) yang dibuang ke tanah akan dengan cepat terurai dan diserap oleh tanah (menjadi bagian dari tanah)
   1. setuju
   2. tidak setuju
   3. tidak tahu
3. Sampah pembungkus makanan dan plastik kemasan lain yang dibuang ke tanah akan dengan cepat terurai dan diserap oleh tanah (menjadi bagian dari tanah)
   1. setuju
   2. tidak setuju
   3. tidak tahu
4. Manakah diantara barang-barang berikut yang boleh dibuang ke tanah? – pilih yang paling sesuai
   1. Puntung rokok
   2. Bungkus/kemasan plastik
   3. Botol plastik kosong
   4. Sampah makanan
   5. Sampah kertas
   6. Tidak tahu
5. Apakah arti “daur-ulang” menurut anda?
6. Mengubah plastik menjadi kerajinan tangan
7. Proses mengolah sampah
8. Tidak tahu
9. Lainnya
10. Pilihan lainnya _________________________________________
11. Manakah diantara barang-barang berikut yang bisa diolah kembali di pabrik? – pilih yang paling sesuai
12. Botol plastik bekas air minum
13. Kaleng minuman soda/coca cola
14. Bekas gelas minuman plastik
15. Bekas bungkus rokok
16. Botol kaca
17. Bungkus/kemasan bekas mie instan
18. Tidak tahu
19. Apa yang harus dilakukan terhadap sampah-sampah plastik (botol, bungkus plastik dan kemasan lainnya) yang tidak bisa dikumpulkan - pilih yang paling sesuai
20. Dibuang ke tempat sampah
21. Ditimbun ke tanah
22. Dibakar
23. Dibuang ke laut
24. Tidak tahu
25. Lainnya ____________________________________________________
26. Apa yang dilakukan lingkungan atau komunitas anda terhadap sampah rumah tangga?
27. Hampir seluruhnya dibuang ke laut
28. Hampir seluruhnya dibakar
29. Hampir seluruhnya dikumpulkan dan dibuang ke tempat sampah/pembuangan
30. Hampir seluruhnya dibiarkan di tanah
31. Tidak tahu
32. Lainnya __________________________________________________
33. Bagaimana cara sampah di rumah tangga anda dibuang?
34. Hampir seluruhnya dibuang ke laut
35. Hampir seluruhnya dibakar
36. Hampir seluruhnya dikumpulkan dan dibuang ke tempat sampah/pembuangan
37. Hampir seluruhnya dibiarkan di tanah
38. Tidak tahu
39. Lainnya __________________________________________________
40. Seberapa jauh anda rela berjalan untuk membuang sampah rumah tangga ke tempat sampah/pembuangan?
    1. Di luar rumah
    2. Jaraknya hanya 1 atau 2 rumah
    3. Jaraknya sampai dengan 5 rumah
    4. Jaraknya sampai dengan 10 rumah
    5. Jaraknya lebih dari 10 rumah
41. Apakah di lingkungan anda terdapat tempat pembuangan sampah bersama?
    1. Ya
    2. Tidak
    3. Tidak tahu
42. Apakah di lingkungan anda menyediakan jasa pengambilan sampah secara berkala (yang disediakan oleh desa atau pemerintah setempat)?
    1. Ya
    2. Tidak
    3. Tidak tahu
43. Apakah di lingkungan anda terdapat “bank” sampah untuk sampah plastik?
    1. Ya
    2. Tidak
    3. Tidak tahu
44. Tempat fasilitas daur-ulang sampah plastik terdekat berlokasi di:
    1. Sekitar lingkungan anda
    2. Kendari
    3. Makassar
    4. Jakarta
    5. Tidak tahu
45. Apakah pengelolaan sampah menjadi permasalahan di lingkungan anda?
    1. Ya
    2. Tidak
    3. Tidak tahu
46. Bagaimanakah pendapat anda mengenai sampah plastik yang ditemukan di pantai/laut
    1. Itu adalah salah satu masalah
    2. Itu bukanlah masalah
    3. Tidak tahu
47. Apa yang terjadi terhadap sampah plastik ketika dikumpulkan?
    1. Akan dibawa ke tempat penimbunan sampah
    2. Akan dibawa ke pembuangan sampah
    3. Akan didaur-ulang menjadi produk plastik lain
    4. Tidak tahu
    5. Lainnya ___________________________________________________
48. Manakah diantara jenis sampah dibawah yang akan mempengaruhi kerusakan laut?
    1. Sampah plastik
    2. Sampah tanaman/dedaunan
    3. Puntung rokok
    4. Sampah dapur rumah tangga
    5. Sampah dari peralatan pancing
    6. Tidak tahu
49. Apakah ikan dan hewan laut lainnya memakan sampah plastik?
    1. Ya
    2. Tidak
    3. Tidak tahu
50. Apakah akibat/dampak yang ditimbulkan sampah plastik terhadap lingkungan?
51. Dampak positif
52. Tidak berdampak apa-apa
53. Dampak negatif
54. Tidak tahu

Bisakah anda mengatakan alasannya?

1. Apakah pembakaran sampah, termasuk pembakaran sampah plastik, mempengaruhi kesehatan manusia?
2. Ya
3. Tidak
4. Tidak tahu

Jika iya, bagaimana pengaruhnya?

1. Menyebabkan batuk
2. Membuat sakit
3. Membuat sulit bernafas
4. Polusi udara
5. Tidak tahu
6. Lainnya ________________________________________________
7. Darimanakah asal sampah plastik yang ada di pantai/laut? - pilih yang paling sesuai
8. Komunitas/lingkungan sendiri
9. Desa lain
10. Kapal/perahu
11. Pulau lain
12. Negara lain
13. Turis, wisatawan
14. Kapal penangkap ikan
15. Lainnya ______________________________________________________
16. Sampah yang terdapat di tanah/daratan akan bermuara di pantai/laut
17. Setuju
18. Tidak setuju
19. Tidak tahu
20. Berapa lama sampah plastik di laut akan bertahan dan terus ada?
21. Sampai dengan 2 hari
22. Sampai dengan 2 minggu
23. Sampai dengan 2 bulan
24. Sampai dengan 20 tahun
25. Sampai dengan 200 tahun
26. Lebih dari 200 tahun
27. Berapa lama sampah botol plastik di laut akan bertahan dan terus ada?
28. Sampai dengan 4 hari
29. Sampai dengan 4 minggu
30. Sampai dengan 4 bulan
31. Sampai dengan 40 tahun
32. Sampai dengan 400 tahun
33. Lebih dari 400 tahun
34. Berapa lama sampah bekas peralatan pancing di lautan akan bertahan dan terus ada?
35. Sampai dengan 6 hari
36. Sampai dengan 6 minggu
37. Sampai dengan 6 bulan
38. Sampai dengan 60 tahun
39. Sampai dengan 600 tahun
40. Lebih dari 600 tahun
41. Pernahkah anda mendengar tentang “mikroplastic”- bagian terkecil dari plastik yang mengapung di lautan?
42. Ya pernah
43. Tidak pernah
44. Tidak tahu
45. Menurut anda, apakah “mikroplastik” memiliki dampak terhadap kesehatan manusia?
46. Dampak positif
47. Tidak berdampak
48. Dampak negatif
49. Tidak tahu
50. Beberapa ilmuwan memprediksi bahwa dalam 7 tahun mendatang, lautan akan dipenuhi plastik daripada ikan
51. Setuju
52. Tidak setuju
53. Tidak tahu
54. Sampah plastik akan menyebabkan turis/wisatawan malas berkunjung ke daerah anda
    1. Setuju
    2. Tidak setuju
    3. Tidak tahu
55. Apakah dampak yang ditimbulkan sampah plastik terhadap pariwisata di daerah anda (sebutkan jika ada)
    1. Dampak positif
    2. Tidak berdampak
    3. Dampak negatif
    4. Tidak tahu

Jika anda menjawab 1 atau 3, jelaskan alasannya:

**Demografis**

1. Gender/jenis kelamin
2. Umur
3. Tingkat pendidikan tertinggi
4. Tidak tamat Sekolah dasar (SD)
5. Tamat Sekolah Dasar (SD)
6. Tidak tamat Sekolah Menengah Pertama (SMP)
7. Tamat Sekolah Menengah Pertama (SMP)
8. Tidak tamat Sekolah Menengah Atas (SMA)
9. Tamat Sekolah Menengah Atas (SMA)
10. Universitas
11. Berapa orang yang tinggal di rumah tangga anda?

- Orang dewasa:
- Anak-anak (dibawah 15 tahun):

1. Berapa lama anda tinggal di lingkungan ini?
2. Kurang dari 10 tahun
3. 10 – 14 tahun
4. 15 – 20 tahun
5. 20 – 24 tahun
6. 25 – 30 tahun
7. Lebih dari 30 tahun
8. Mohon perkirakan total pemasukan rumah tangga anda (rata-rata per minggu)

(kategorinya sebagai berikut):

1. < Rp 300,000
2. Rp 301,000 - Rp 1,000,000
3. Rp 1,000,001 - Rp 2,000,000
4. Rp 2,000,001 - Rp 3,000,000
5. Rp 3,000,001 - Rp 4,000,000
6. Rp 4,000,001 - Rp 5,000,000
7. > Rp 5,000,000
8. Apakah mata pencaharian anda?
9. Nelayan
10. Ibu rumah tangga
11. Petani
12. Lainnya

____________________________________
