# Supplementary material for: Ocean plastic crisis—Mental models of plastic pollution from remote Indonesian coastal communities
Source: PLoS One. 2020 Jul 28;15(7):e0236149. doi: 10.1371/journal.pone.0236149 (PMC7386615; doi:10.1371/journal.pone.0236149)
Supplement: S2 Data — (DOCX) [file pone.0236149.s002.docx]

**Project:** SMALL ISLANDS INITIATIVE FOR A PLASTIC FREE OCEAN

***Date: Respondents Code
(Name of Enumerator; respondent no):***

***Village: Sub-village (if applicable):***

Household position

|  | - 1. Near the beach |
| --- | --- |
|  | - 1. Centre of village |
|  | - 1. Back of village |

1. What are the biggest problems that you face in your community?
2. Organic waste (e.g. food waste, plant litter) thrown on the ground will quickly break down and disappear (become part of the soil)

| 1 | Agree |
| --- | --- |
| 2. | Disagree |
| 3. | Not sure |

1. Snack food wrappers and other plastic packaging thrown on the ground will quickly break down or disappear

| 1 | Agree |
| --- | --- |
| 2. | Disagree |
| 3. | Not sure |

1. Which of the following items is ok to throw (discard) on the ground? – choose all that apply
   1. cigarette butts
   2. plastic packaging
   3. empty plastic bottles or containers
   4. food waste
   5. waste paper
   6. not sure
2. What does ‘recycle’ mean to you?
3. Turning plastic into handicrafts
4. Rubbish processing
5. Not sure
6. Other
7. Other Text _________________________________________
8. Which of the following items can be re-processed in a factory – choose all that apply
9. Plastic water bottle
10. Coca-cola can
11. Plastic water ‘glass’
12. Empty cigarette pack
13. Glass bottle
14. empty instant noodle packet
15. Not sure
16. Plastic waste (bottles, wrappers and other packaging) that cannot be collected should be: (choose all that apply)
17. Put in the bin
18. Buried
19. Burned
20. Put in the ocean
21. Not sure
22. Other

___________________________________________________

1. What happens to household waste in your community?
2. Most of it is thrown in the ocean
3. Most of it is burned
4. Most of it is collected and put in a collection area/bin
5. Most of it remains on the ground
6. Not sure
7. Other

__________________________________________________

1. How does your household dispose of waste?
2. Most of it is thrown in the ocean
3. Most of it is burned
4. Most of it is collected and put in a collection area/bin
5. Most of it remains on the ground
6. Not sure
7. Other

_______________________________________________

1. How far would you be prepared to walk to dispose of your household’s daily rubbish in a bin?
   - 1. Outside your house
     2. One or two houses away
     3. Up to 5 houses away
     4. Up to 10 houses away
     5. More than 10 houses away
2. Does your community have a rubbish collection point?

|  | 1. Yes |
| --- | --- |
|  | 1. No |
|  | 1. Not sure |

1. Does your community have regular waste collection services provided by the village or local government?

| 1. Yes |
| --- |
| 1. No |
| 1. Not sure |

1. Does your community have a garbage/waste ‘bank’ for plastic waste?

|  | 1. Yes |  |
| --- | --- | --- |
|  | 1. No |  |
|  | 1. Not sure |  |

1. The closest recycling facility (plastic processing factory) is located in:
   - 1. Your village
     2. Ambon / Kendari
     3. Makassar
     4. Jakarta
     5. Not sure
2. Is waste management a problem for your community?

| 1. | Yes |
| --- | --- |
| 2. | No |
| 3. | Not sure |

1. What do you think about plastic found in the ocean and on the beach

| 1. | It is a problem |
| --- | --- |
| 2. | It is a not problem |
| 3. | Not sure |

1. What happens to plastic waste after it is collected?
   - 1. It is taken to a landfill
     2. It goes to a garbage bank
     3. It is recycled into plastic products
     4. Not sure
     5. Other

__________________________________

1. Do any of the following types of rubbish affect marine life?
   1. Plastic litter
   2. Leaf litter
   3. Cigarette butts
   4. Kitchen waste
   5. Discarded fishing line
   6. Not sure
2. Do fish and other marine animals eat plastic waste?

| 1. | Yes |
| --- | --- |
| 2. | No |
| 3. | Not sure |

1. What effect does plastic waste have on the environment?

| 1. | Positive effect |
| --- | --- |
| 2. | No effect |
| 3. | Negative effect |
| 4. | Not sure |

Can you tell me why you think that? ___________________

1. Does burning rubbish, including plastic, affect human health?

| 1. | Yes |
| --- | --- |
| 2. | No |
| 3. | Not sure |

If ‘Yes’, how does it affect human health?

1. Causes coughing
2. Makes you sick
3. Makes it difficult to breathe
4. Air pollution
5. Not sure
6. Other

______________________________________

1. Where does plastic found on the beach come from? – choose all that apply
2. Our community
3. Other villages
4. Boats and ferries
5. Other islands
6. Other countries
7. Tourists, visitors
8. Fishing boats
9. Other
10. Rubbish left on the ground will eventually make its way into the ocean

| 1. | Agree |
| --- | --- |
| 2. | Disagree |
| 3. | Not sure |

1. In the ocean, how long does the plastic that makes up a plastic bag last?
2. Up to 2 days
3. Up to 2 weeks
4. Up to 2 months
5. Up to 20 years
6. Up to 200 years
7. More than 200 years
8. In the ocean, how long does the plastic that makes up a plastic bottle last?
9. Up to 4 days
10. Up to 4 weeks
11. Up to 4 months
12. Up to 40 years
13. Up to 400 years
14. More than 400 years
15. In the ocean, how long does discarded fishing line does last?
16. Up to 6 days
17. Up to 6 weeks
18. Up to 6 months
19. Up to 60 years
20. Up to 600 years
21. More than 600 years
22. Have you heard about ‘microplastics’ - tiny pieces of plastic floating in the ocean?

| 1. | Yes |
| --- | --- |
| 2. | No |
| 3. | Not sure |

1. What effect do you think these ‘microplastics’ might have on human health

| 1. | Positive effect |
| --- | --- |
| 2. | No effect |
| 3. | Negative effect |
| 4. | Not sure |

1. Some scientists estimate that in seven years the oceans may contain more plastic than fish

| 1. | Agree |
| --- | --- |
| 2. | Disagree |
| 3. | Not sure |

1. Plastic waste will discourage some tourists from visiting your community

| 1. | Agree |
| --- | --- |
| 2. | Disagree |
| 3. | Not sure |

1. What effect (if any) might plastic waste have on tourism in your community?

| 1. | Positive effect |
| --- | --- |
| 2. | No effect |
| 3. | Negative effect |
| 4. | Not sure |

For answers 1 & 3, please explain____________________________________

**Demographics**

1. Gender
2. Male
3. Female
4. Age
5. What is your highest level of education?

| 1. | Primary education (not completed) |
| --- | --- |
| 2. | Primary education completed |
| 3. | Middle school (not completed) |
| 4. | Middle school completed |
| 5. | High school (not completed) |
| 6. | High school completed |
| 7. | University |

1. How many people living in your household?

- Adults
- Children (under 15)

1. How long have you been living in this community?
2. Less than 10 years
3. 10 – 15 years
4. 15 – 20 years
5. 20 – 25 years
6. 25 – 30 years
7. More than 30 years
8. Please estimate your total household’s weekly income (or weekly average):
9. < Rp 300,000
10. Rp 301,000 - Rp 1,000,000
11. Rp 1,000,001 - Rp 2,000,000
12. Rp 2,000,001 - Rp 3,000,000
13. Rp 3,000,001 - Rp 4,000,000
14. Rp 4,000,001 - Rp 5,000,000
15. > Rp 5,000,000
16. What is your occupation?
17. Fisherman
18. Housewife
19. Farmer
20. Other

____________________________________
